# A Meta-Epidemiological Study of Positive Results in Clinical Nutrition Research: The Good, the Bad and the Ugly of Statistically Significant Findings

**DOI:** 10.3390/nu14235164

**Published:** 2022-12-04

**Authors:** Konstantinos Gkiouras, Maria-Eleftheria Choleva, Aikaterini Verrou, Dimitrios G. Goulis, Dimitrios P. Bogdanos, Maria G. Grammatikopoulou

**Affiliations:** 1 Department of Rheumatology and Clinical Immunology, Faculty of Medicine, School of Health Sciences, University of Thessaly, Biopolis, 41110 Larissa, Greece; 2 Department of Nutritional Sciences and Dietetics, Faculty of Health Sciences, International Hellenic University, Alexander Campus, 57400 Thessaloniki, Greece; 3 Unit of Reproductive Endocrinology, 1st Department of Obstetrics and Gynecology, Medical School, Faculty of Health Sciences, Aristotle University of Thessaloniki, 56403 Thessaloniki, Greece

**Keywords:** nutrition methodology, nutrition epidemiology, publication bias, research methodology, *p* value, meta-analysis, Bonferroni correction, reporting guidelines, research bias

## Abstract

Positive (statistically significant) findings are easily produced in nutrition research when specific aspects of the research design and analysis are not accounted for. To address this issue, recently, a pledge was made to reform nutrition research and improve scientific trust on the science, encompass research transparency and achieve reproducibility. The aim of the present meta-epidemiological study was to evaluate the statistical significance status of research items published in three academic journals, all with a focus on clinical nutrition science and assessing certain methodological/transparency issues. All research items were published between the years 2015 and 2019. Study design, primary and secondary findings, sample size and age group, funding sources, positivist findings, the existence of a published research protocol and the adjustment of nutrients/dietary indexes to the energy intake (EI) of participants, were extracted for each study. Out of 2127 studies in total, those with positive findings consisted of the majority, in all three journals. Most studies had a published research protocol, however, this was mainly due to the randomized controlled trials and not to the evidence-synthesis studies. No differences were found in the distribution of positive findings according to the existence/inexistence of a published research protocol. In the pooled sample of studies, positive findings differed according to study design and more significant findings were reported by researchers failing to report any funding source. The majority of items published in the three journals (65.9%) failed to account for the EI of participants. The present results indicate that there is still room for the improvement of nutrition research in terms of design, analyses and reporting.

## 1. Introduction

For years, the interaction between foods, nutrients and disease was modeled on the basis of linear relationships and associations, forming recommendations that often, were later found to be flawed, or even inverse. The need for evidence-based nutrition and more “unbiased” interactions between nutrition and health have helped the science evolve [1,2,3]. This resulted in abandoning the Bradford-Hill criteria [4], while embracing the pyramid of evidence [5] following the contemporary model of cause and effect, as in the rest of health and medical sciences.

Nevertheless, nutrition research has often been questioned for “cherry picking” significant findings [6], being pedaled by the industry [7,8,9] or for failing to follow the rules of nutritional epidemiology for correcting common nutrition bias [10,11]. For instance, the Hawthorne effect [12], an indisputable component of all nutrition research, can be diminished by distinguishing the participants to adequate-, over- or low-energy reporters [13,14]. Under-reporting of dietary intake consists of a fundamental problem in nutrition epidemiology, inducing bias in the habitual dietary intake data [15]. In parallel, as far as nutrients are concerned, given that their intake is greatly dependent on the total energy consumption of the participants in a study [16], with greater-energy consumers always exhibiting an increased intake in all nutrients, a correction of all intakes based on the level of energy intake (EI) is deemed necessary to balance the findings [16,17,18]. Similarly, dietary indexes based on nutrients are also depended on the EI, and, according to Willet [16,17], should also be adjusted to the EI of the participants. Furthermore, to avoid “cherry picking” of significant results, study outcomes are predefined and published using a protocol. Primary outcomes are variables with the most evidence of being associated with the intervention or the exposure of interest, and are thus, highly relevant to answer the research hypothesis, ideally, from the patient’s perspective [19,20]. On the other hand, usually the secondary endpoints consist of additional outcomes “measured” to help us interpret the results of the primary endpoints, and their selection requires justification [19,20]. Unfortunately, due to its nature, most of nutrition research including dietary analysis uses many outcomes, and this can often result in unfocused research questions and difficulty in interpretating the effect of treatment [20], or the exact role of each nutrient.

Collectively, it all points to the fact that positive (statistically significant) findings, based on the null-hypothesis testing, are easily produced in nutrition research [3,6] when specific aspects of research design and analysis are not accounted for. To address this issue recently, a pledge was made to reform nutrition research and improve scientific trust on the science, encompass research transparency and achieve reproducibility of the findings [2,3,10,11,21,22]. Nonetheless, we are not sure to what extent good practices are followed, even among the most high-impact clinical nutrition journals. In this manner, the aim of the present meta-epidemiological study was to evaluate the statistical significance status of the research items published in three academic journals, all with a focus on the science of clinical nutrition and to assess certain methodological/transparency issues.

## 2. Materials and Methods

### 2.1. Search Strategy

The full-text articles of three academic journals with clinical nutrition as their main subject were searched from the year 2015 until the end of 2019, by two researchers independently (M-E.C. and A.V.). When disagreement was apparent, a more senior researcher would step in to aid in coming to a consensus (M.G.G.). Three academic journals dedicated to the science of nutrition were selected, each with different impact factor (IF). One of the journals had attained a high IF (*American Journal of Clinical Nutrition, AJCN*), one had a moderate IF (*European Journal of Clinical Nutrition, EJCN*) and the last journal had a lower IF (*Clinical Nutrition ESPEN, Clin Nutr ESPEN*) at the time of the study.

Published manuscripts in the aforementioned journals were retrieved using the published electronic issue catalogues, listed on each journal’s website.

### 2.2. Inclusion and Exclusion Criteria

Published studies were included in the analyses provided they (a) had a hypothesis-driven analysis for primary or secondary outcomes, based on the level of statistical significance (*p*), (b) were performed on human samples irrespective of their age, (c) had a primary evidence design, including longitudinal studies (cohort, retrospective or prospective), cross-sectional (including case-control or diagnostic accuracy studies) or clinical trials (randomized controlled trials (RCTs) and non-RCTs), or d) had a meta-research (MR) design, including systematic reviews (SRs), SRs with meta-analysis (MA), network MA (NMAs) or other MR (umbrella reviews, and meta-epidemiological studies).

Manuscripts were excluded when they involved (a) studies performed on animals, or preclinical studies, (b) basic science research, (c) letters to the editor without primary data, (d) narrative or scoping reviews without the production of primary or secondary data, (e) clinical practice guidelines or consensus, (f) simulation or modeling studies, (g) qualitative research which inevitably does not use a statistical significance threshold and (h) studies lacking hypothesis testing.

### 2.3. Data Extraction

Microsoft excel was used for the extraction of data from the retrieved research items. Information regarding the journal, type of study (narrative review, SR, MA, NMA, RCT, cross-sectional, cohort, non-RCT, etc.), registration information (for RCTs, MAs and NMAs) including registry, journal-based protocol publication and/or registration number, funding declaration and country of origin were extracted for each manuscript by two independent reviewers (M.-E.C., A.V.). When differences arose between researchers regarding a research item, a more senior researcher resolved any conflict by discussion. Funding declaration, as well as primary and secondary funding sources (industrial, academic or by scientific/health organizations) were also recorded. When multiple funding sources were reported, the first was selected for each research item in the following order industrial-organizational-academic.

For each original research item (with a cross-sectional, cohort or clinical trial design), the a priori classification of the study endpoints as primary or secondary, and the statistical significance of primary and secondary findings were recorded. The age group of the population used in each study was also recorded (adult, non-adult or not defined), in parallel to the possible use of dietary indexes and their adjustment, or lack of adjustment according to the energy intake of participants. Sample size was also recorded, and when intention-to-treat analyses were performed, this sample was used for the analyses. Moreover, the statistical significance of the findings (*p* value) was also recorded for each research item. When energy intake (EI) data were reported, the classification of the sample to adequate-, over- or under-reporters, as well as the equations/methods used to calculate the energy requirements, were further extracted. In the case of dietary indexes, adjustment to the EI of participants was also recorded.

### 2.4. Stratification of Results Based on the Statistical Significance Level

The *p* values of all primary and secondary endpoints were categorized based on the existence of a statistical significance or not for each comparison. When the primary or secondary endpoints were not defined in the study publications or protocols (when available), this information was sought within the manuscript text of each publication. In the cases when more than one outcome was defined in each category (primary/secondary), the first reported primary or secondary outcomes were considered as the main ones for that study. When the exact *p* values were not presented, the stratification of findings according to the statistical significance was performed based on whether the results indicated a significance or if this was reported in the manuscript text.

*p* values were categorized based on the α = 0.05 cutoff (95% confidence intervals, CI) and on whether researchers used adjustments to the *p* values (e.g., Bonferroni correction). When the independent variables had multiple categories (e.g., different tiers of nutrient intake), the *p* values of the last category were recorded given that it is common to compare the last and first groups, according to Ioannides [6]. When the last group was the reference group, then the *p* value of the first group was recorded. Emphasis was given to the analyses accounting for a greater number of confounders, as such analyses are often the basis for extrapolating findings. Furthermore, when independent variables were presented as categorical and quantitative ones, emphasis was given to the quantitative variables, as per Altman [23,24]. Furthermore, emphasis was given to the analyses using the total sample of the study, but when subgroups of the sample also demonstrated statistical significance, these values were also recorded. When multiple scales were used to “measure” the dependent variable without any information being provided on which was more valid than the others, the analyses with statistically significant findings were selected. When *p* values were not presented in the manuscript text, but the studies were basing their findings on a certain degree of significance (e.g., α = 0.05), then the results were categorized as significant or not, based on the 95% CI. For example, mean differences with 95% CI crossing the zero were considered as non-significant. When the studies were presenting their results as an effect size, without considering significance levels, these were not considered in the analyses. Similarly, studies lacking the report of *p* values and 95% CI were also excluded.

### 2.5. Data Analyses

The results were presented as frequencies and their respective proportions in parentheses. The chi-square test was employed to test for differences in the distribution of proportions. In the cases when more than two categories existed in a qualitative variable, then no conclusion could be drawn from the chi-square test regarding their difference. In these cases, the chi-square test was followed by post-hoc analyses, with the Bonferroni correction. For example, if five comparisons were performed within the five distinct categories of one variable, the Bonferroni-adjusted *p* value that was considered as significant would be α = 0.05/4 = 0.0125 [25]. The 0.05 value was considered as a cutoff for the significance level in all chi-square tests, the 0.0125 value was selected for comparisons between four categories (comparisons between study types) and the 0.0083 value was the cutoff for comparisons involving six categories (analyses according to the funding source). The six post-hoc comparisons based on the funding source of each study included: (a) funding from academic, industry and organizations versus no disclosed funding, or (b) funding from academic, industry and organizations versus no funding.

Regarding the analyses according to the sample size, post-hoc comparisons were performed between sample populations whose age was not defined, versus all other age groups. Thus, multiple comparisons did not exist, and Bonferroni corrections were not performed. All analyses were performed in SPSS version 25.0 (SPSS, Chicago, IL, USA).

## 3. Results

### 3.1. Search Results

**Figure 1:**
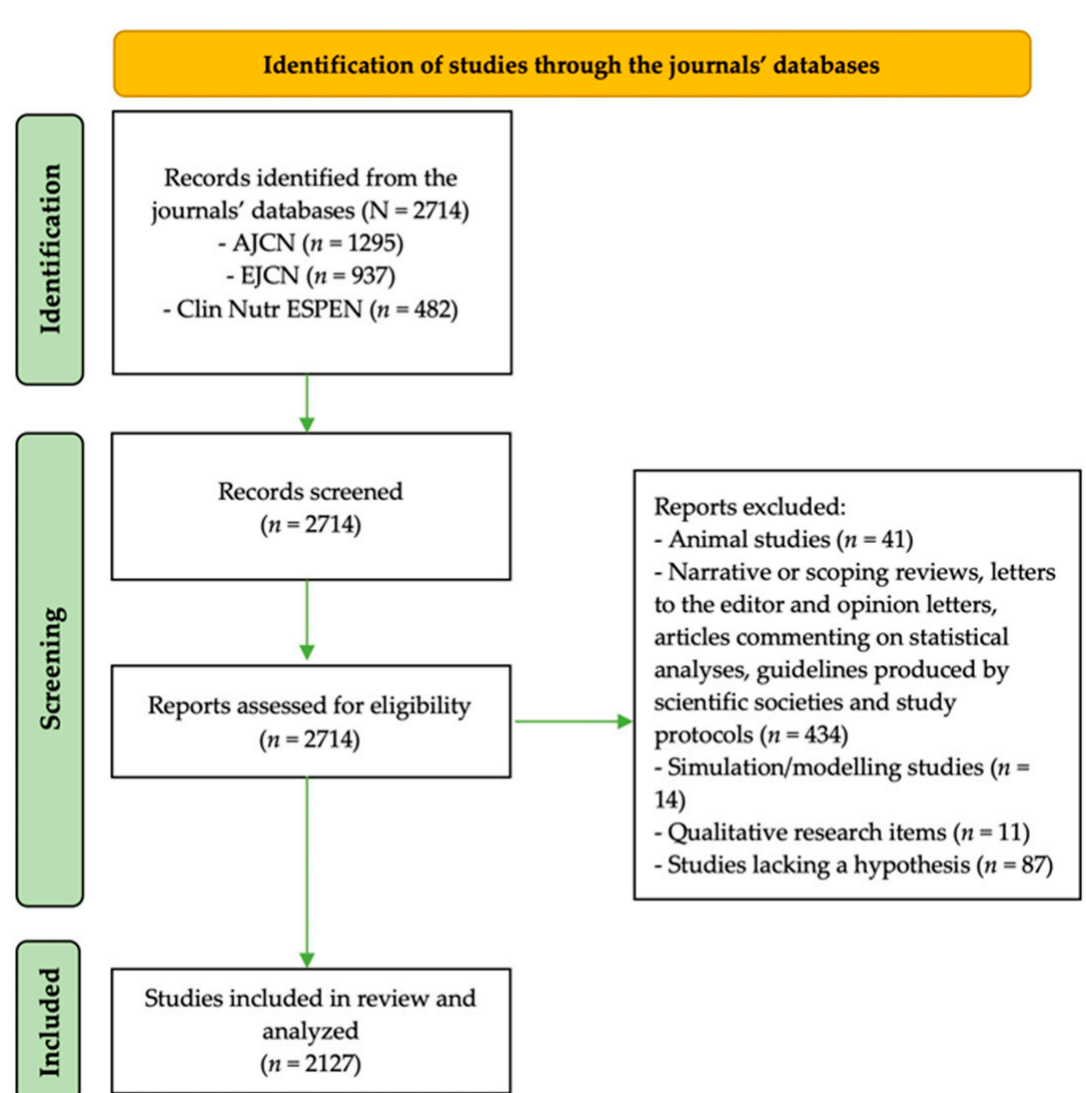
Flow-chart of the studies selection process. AJCN, American Journal of Clinical Nutrition; Clin Nutr ESPEN, Clinical Nutrition ESPEN; EJCN, European Journal of Clinical Nutrition.

A total of 2714 full-text articles were retrieved, 1295 published in the *AJCN*, 937 in the *EJCN* and the remaining 482 in the *Clin Nutr ESPEN* journal. After screening the articles for the exclusion of the items not fulfilling the study’s criteria, 41 animal studies, 434 narrative or scoping reviews, letters to the editor and opinion letters, articles commenting on statistical analyses, guidelines produced by scientific societies and study protocols, 14 simulation/modeling studies, 11 qualitative research items and 87 studies lacking a hypothesis were excluded. Thus, the final sample included a total of 2127 studies (Figure 1).

### 3.2. Significant Findings

**Figure 2:**
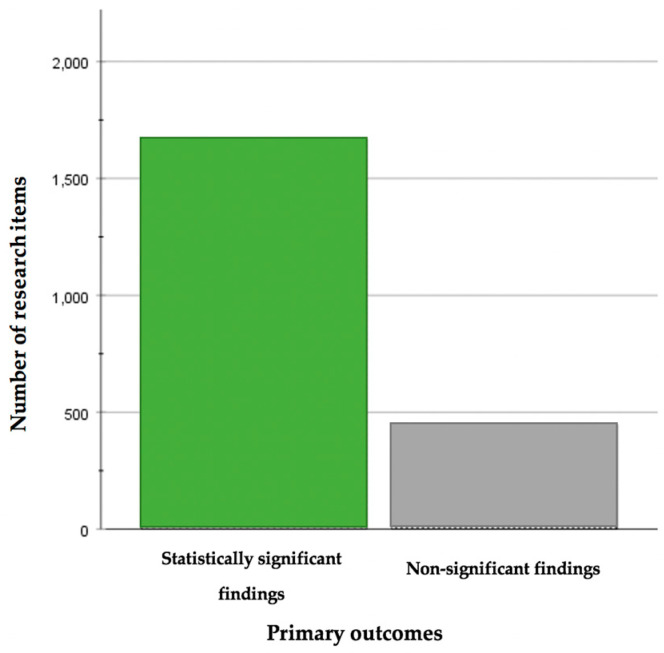
Histogram presenting the distribution of statistically significant and non-significant findings in all three academic journals.

Overall, the published studies with significant findings consisted of the majority in all three journals (Figure 2, Table 1). The chi-square test revealed that with regard to both the primary (*p* < 0.001) and the secondary outcomes (*p* = 0.029), distinct study designs produced findings of different statistical significance. In particular, post-hoc analyses showed that with regard to the primary endpoints, RCTs (*p* = 4 × 10^−6^) and cross-sectional/non-RCT studies differed (*p* = 0.007) from the rest of the study designs, whereas with regard to the secondary outcomes, studies adopting a cross-sectional/non-RCT design differed from the remaining research items (*p* = 0.003).

Within individual journals, positive outcomes did not differ in any of the study designs adopted by the research items published in the *AJCN*. On the other hand, different study designs produced different findings in terms of statistical significance in the *EJCN* (*p* = 0.001). In particular, SRs/MAs or MR (including NMAs) articles published in the *EJCN* differed from the rest of the study designs in terms of positive findings (*p* = 0.004). Finally, with regard to the *Clin Nutr ESPEN* journal, the chi-squared test showed that the study designs affected the production of positive findings in both the primary (*p* = 0.003) and secondary (*p* = 0.002) outcomes. In further detail, RCTs differed with regard to the primary and secondary outcomes and their positive findings (*p* = 2 × 10^−4^ and *p* = 0.005, respectively).

### 3.3. Positive Findings according to Funding Sources

Table 2 details the distribution of significant findings for each type of funding source. In all three journals, the majority of publications reported receiving funding from organizations (56.9%, *p* = 0.001).

Post-hoc analyses revealed that within the main outcomes, more positive findings were reported within the studies failing to report any funding source (*p* = 5.7 × 10^−4^), as compared to those receiving funding from the academia. The same analyses also showed that more significant findings were reported among studies lacking funding, compared to those funded by the industry (*p* = 0.002). No differences were noted in the statistical significance of the secondary outcomes by funding source (*p* = 0.259).

In the *EJCN*, differences were noted in the level of significance of the primary outcomes by distinct funding sources (*p* = 0.036), however, post-hoc analyses did not identify any specific funding source as more prone to positive findings. In the *Clin Nutr ESPEN* journal, although differences were noted in the level of significance of the primary outcomes between funding sources (*p* = 0.022), none of the post-hoc analyses identified further discrepancies.

Table 3 details the results of significant findings according to secondary funding source. In the pooled journals sample, the secondary outcomes differed in terms of significant findings (*p* = 0.002). Post-hoc analyses revealed that the research items not reporting any funding source presented more positive findings compared to the studies being funded by organizations (*p* = 8.7 × 10^−6^).

### 3.4. Positive Findings according to Sample Size

Table 4 details the positive findings of the research items according to the age group of the participating populations in each study.

The majority of studies used adult participants (77.6%) in their samples. Among the research items published in the *AJCN* that year, the positive findings regarding the secondary outcomes differed between the distinct participant age groups (*p* = 0.020). In further detail, post-hoc analyses revealed that the proportion of positive findings was smaller in unidentified participant age groups, compared to the rest of the age groups (*p* = 0.033). As far as studies published in the *Clin Nutr ESPEN* journal were concerned, although positive findings differed between distinct age groups (*p* = 0.048), the post-hoc analyses failed to identify specific difference pairs.

### 3.5. Positive Findings among Studies with a Published Protocol

The statistically significant findings of the studies are presented in Table 5 based on whether the study had a pre-published research protocol, or not. Among the pooled research items from all three journals, it was observed that the majority of studies had a published research protocol, however, this was mainly due to the RCTs and not to the SRs/MAs/MR studies. The chi-squared tests failed to reveal any differences in the distribution of positive findings according to the existence/inexistence of a published research protocol. 

### 3.6. Positive Findings in Studies Adjusting for the Energy Intake of Participants

Table 6 details the distribution of positive findings based on the EI adjustment. The majority of items published in the three journals combined (65.9%) failed to account for the total EI of participants. The chi-squared test revealed that cohort studies where the EI adjustment was not accounted for, exhibited a greater proportion of positive findings in their primary outcomes (*p* = 0.006).

## 4. Discussion

The present study reveals that nutrition research design, analysis and reporting have room for improvement in order to improve scientific quality and reporting transparency. Most of the examined clinical nutrition research items had positive findings and failed to account for the EI of the participants. Reports of studies not identifying any funding source were more likely to produce positive findings. Moreover, positive findings differed according to study design.

### 4.1. Adjusting for the EI of Participants in Nutrition Research

In the present study, the majority of research items failed to account for the total EI of participants. As per Willet [16,17], adjustment of the intake and calculation of dietary indexes for the total EI of the sample is required in epidemiologic studies, in order to control for possible confounding, limit variation due to extraneous consumption and predict more accurately the effect of dietary interventions. On the other hand, failure to control for total EI can erroneously shift the direction of associations between nutrient intakes and the risk of developing disease [16,17], as promptly noted by Ioannidis [6]. In Layman’s terms, adjustment for total EI is required for the same reasons that diets of a specific isocaloric content are used in experiments, in order to evaluate the effects of specific nutrients [17]. Of course, participants who consume a greater quantity of energy, will inevitably also consume a greater overall quantity of most nutrients. In a relevant analysis, Rhee [26] showed that energy adjustment calculated from the same questionnaire used to estimate nutrient intakes can greatly improve the correlation of some nutrients with their biomarkers. Lack of EI adjustment can be an important confounding factor for epidemiologic studies, and this should be kept in mind when designing and executing nutrition research.

In a comprehensive manner, Tomova [18] detailed four distinct models that can be applied to correct for this error. The first is the “standard model”, where adjustment is based on the total EI of participants, the second involves the “energy partition model”, where the adjustment is performed using the remaining EI, corresponding to the calories consumed from all sources excluding the nutrient of interest [18]. The third method is the “nutrient density model”, in which the nutrient exposure is examined as a proportion of the total EI and the forth approach consists of the “residual model”, where adjustment is performed for the residual produced by regressing the nutrient exposure on total EI [18]. Nonetheless, an additional method has been proposed as a better estimate of the energy intake compared to food frequency questionnaires or food diaries, involves the adjustment for body mass and physical activity levels [27]. However, this method has also been judged for being imperfect, since it may also involve additional measurement errors [28]. Moreover, in a relevant comparison, Rhee and associates [26] showed that the residual method was superior to the one involving adjustment based on the body weight and physical activity, thus this approach was quickly abandoned. 

### 4.2. Nutrition Research and Industry Funding

Recently, concerns have been raised regarding the role of the industry in the funding and conduction of nutrition research [9,29]. Industry funding has been deemed as damaging for the integrity of nutrition research [29,30,31], as several companies appear to purposefully influence the research design [32,33,34]. Using publication-level data, Rao [31] showed that compared to non-industry funded research, industry-funded studies report 3.2% more positive findings, with grains in particular, being responsible for most of the observed effect, as they are being funded in a greater proportion by the industry. Fabbri [34] examined the research funded by two major food industries, namely Coca-Cola and Mars, and revealed that research sponsored by these companies appeared “to skew the evidence towards solutions favoring industry interests”, indicating commercial bias in the research agenda. In an analysis of original research articles published in the year 2018 in the highly cited journals with a nutrition and dietetics scope, a total of 13.4% of the articles reported the involvement of the food industry, with the highest rate being observed at *The Journal of Nutrition* (28.3%) [35]. Interestingly, most of the articles involved processed food manufacturers (39.3%) and of all the articles reporting involvement of the food industry, 55.6% reported favorable findings to the food industry’s interests, compared to 9.7% of the research items without any food industry involvement [35].

What is more concerning is that this agenda often goes beyond the publication of a research item in a scientific journal or conference. Understanding the strength of the associations between nutrients and disease risk is the basis for the development of population-level recommendations [36]. Lauber and associates [37] identified all claims made from organizations/lobbies representing ultra-processed food industry (UPFI) companies to World Health Organization consultations regarding the formation of policy for non-communicable diseases (NCDs), between the years 2016 and 2018. Out of a total of 114 claims made, 56 of those were only backed-up by 39 relevant research items [37]. Additionally, among these 39 pieces of evidence, two-thirds were industry-linked or directly industry-funded, and only 16 were in fact, peer-reviewed externally [37]. Similar issues have also been reported with regard to the “Eatwell guide” [38] and the Dietary Guidelines for Americans (DGAs), with several scientists asking for more transparency in the conflicts of interest (COIs) of the DGA advisory committee [30,39,40,41,42,43]. However, at the level of individual scientists/academics, a paradox exists at the moment: although universities crave funding from any source, they hold individuals responsible for managing COIs, covering up the systemic problems that develop from institutional partnerships with the industry [44]. Suffice to say, industry funding is not necessarily problematic or biased, nonetheless, reporting transparency is required to weigh the involvement of funding in any research.

According to the present study, the majority of published research on clinical nutrition is being funded by scientific organizations rather than the industry, and luckily, no differences were noted in the proportion of positive findings between the industry and other funding sectors. Whether this means that scientists are beginning to understand the role of the industry, if this is particular to the “clinical nutrition” research domain, or if this is coincidental, it is not known, as this was a cross-sectional study.

### 4.3. Publication of the Research Protocol and Adherence to the Reporting Guidelines

The need for specific standards for reporting nutrition research was apparent early on in the scientific community [45]. The publication of research protocols prior to the commencement of a study is considered as an important parameter of research transparency, limiting selective reporting bias, regardless of the scope of the study. Nonetheless, it appears that not all nutrition studies, whether primary or evidence synthesis, adhere to the protocol publication standard. As far as RCTs are concerned, the CONsolidated Standards of Reporting Trials (CONSORT) statement [46] requires registering and reporting the research protocol in a registry. Similar standards also exist for SRs and MAs [47]. Furthermore, specific standards have been proposed for observational nutrition research based on the Strengthening the Reporting of Observational Studies in Epidemiology-Nutritional Epidemiology (STROBE-nut) guidelines [48]. Nonetheless, even when protocols have been published, they are not always followed, and frequently, as seen in the case of vitamin intervention trials [7], they fail to post their results promptly, possibly due to negative findings. Recent research showed that poor reporting quality is also extended in basic nutrition research performed on mice, where the majority of scientists appear oblivious to the reporting checklists for animal research [49]. Even meta-analyses of nutrition-related research often fail to adhere to the reporting standards for meta-analyses [50]. When funding sources were examined as a possible factor associated with poor reporting of nutrition research, Myers [51] failed to show differences between industry-sponsored research and government-funded projects.

An important question arising from the present work, lays on whether academic journals are responsible for specific “inadequacies” present in nutrition research. As per Altman [52], the misuse of statistics and their reporting is unethical and should be avoided, however, most scientists and statisticians involved in nutrition research appear unaware of all the prerequisites needed to produce more robust findings. According to the International Committee of Medical Journal Editors (ICMJE) [53], one of the responsibilities of journal editors is to ensure that published research is of the best possible quality standards and scientific integrity [54]. In reality though, a great load lays on the peer review process. In this manner, the responsibility for attaining research integrity in nutrition research is segmented between scientific and statistical editors and scientists, including peer reviewers, authors and statisticians working in the field.

A great number of scientists have expressed skepticism for the results of RCTs implementing nutrition interventions [2,55,56,57]. Concerns exist mainly over blinding and the placebo effect, as diet is difficult to blind and specific expectations are inevitably produced either as a placebo, or a nocebo effect. Furthermore, the burden enforced on participants enrolled in nutrition RCTs is great, and the risk for non-adherence increases, the longer a trial continues [58]. For this, the conduction of pragmatic nutrition trials, even with negative findings has been suggested as an alternative [55,59], and specific guidelines were produced to aid nutrition researchers to upscale their study design and reporting [60,61]. Furthermore, the Hierarchies of Evidence Applied to Lifestyle Medicine (HEALM) approach can also be applied to evaluate the evidence strength on associations between dietary patterns and other outcomes [62]. According to Brown [11], what was considered as good enough in the past, is not adequately good at the moment, and more rigorous methodology and reporting are required to promote the science of nutrition.

### 4.4. Study Limitations

Limitations of the present study include the search in three academic journals only, all published by well-known publishing houses. It is possible that an extended search in other journals, including predator journals, might have changed the findings presented herein. Furthermore, due to the great number of research items retrieved, the variety in the nutrients examined in the present analyses and their combinations, subgroup analyses of different nutrients, or comparisons between nutrients and food patterns and their effect on positive findings was not a feasible option. However, it consists of an important issue that could be examined in the future.

## 5. Conclusions

The present study revealed that nutrition research can greatly benefit from the adoption of more rigorous research methodologies and improved reporting. As in all fields, researchers involved in nutrition are greatly responsible for the integrity, transparency and reproducibility of the science and ought to make steps towards the improvement of the design, analyses and reporting of their studies. We should not forget that with regard to population health, nutrition research is competing against big pharma [2]. Although this might increase the scrutiny against nutrition, it can also serve as an additional motive to implement the best and most up-to-date design standards and reporting guidelines to support the science of nutrition in a seemingly uneven war. As this is an exciting time for nutrition research, the findings herein provide food for thought and indicate some areas of nutrition research that can be improved.

## Figures and Tables

**Table 1:** Studies retrieved from each journal and statistically significant findings with regard to their primary and secondary outcomes.

Study Type	N	Positive Outcomes			
		Primary	*p* Value	Secondary	*p* Value
**All journals**	2127	1667		1440	
SRs/ΜAs/ΜR	210 (9.9%)	152 (9.1%)	<0.001	125 (8.7%)	0.009
RCTs	587 (27.6%)	428 (25.7%)		389 (27.0%)	
Cohorts	649 (30.5%)	531 (31.9%)		443 (30.8%)	
Cross-sectional/non-RCTs	681 (32.0%)	556 (33.4%)		483 (33.5%)	
***AJCN***	1020	786		676	
SRs/ΜAs/ΜR	104 (10.2%)	77 (9.8%)	0.234	64 (9.5%)	0.710
RCTs	386 (37.8%)	288 (38.6%)		254 (37.6%)	
Cohorts	323 (31.7%)	258 (32.8%)		215 (31.8%)	
Cross-sectional/non-RCTs	207 (20.3%)	163 (20.7%)		143 (21.2%)	
***EJCN***	720	561		474	
SRs/ΜAs/ΜR	74 (10.3%)	48 (8.6%)	0.001	42 (8.9%)	0.269
RCTs	122 (16.9%)	85 (15.2%)		83 (17.5%)	
Cohorts	229 (31.8%)	187 (33.3%)		146 (30.8%)	
Cross-sectional/non-RCTs	295 (41.0%)	241 (43.0%)		203 (42.8%)	
***Clin Nutr ESPEN***	387	320		290	
SRs/ΜAs/ΜR	32 (8.3%)	27 (8.4%)	0.003	19 (6.6%)	0.002
RCTs	79 (20.4%)	55 (17.2%)		52 (17.9%)	
Cohorts	97 (25.1%)	86 (26.9%)		82 (28.3%)	
Cross-sectional/non-RCTs	179 (46.3%)	152 (47.5%)		137 (47.2%)	

AJCN, American Journal of Clinical Nutrition; Clin Nutr ESPEN, Clinical Nutrition ESPEN; EJCN, European Journal of Clinical Nutrition; MA, meta-analysis; MR, meta-research; RCT, randomized controlled trial; SR, systematic review.

**Table 2:** Positive findings in the primary and secondary outcomes of studies according to the main funding source.

Funding Type	N	Outcomes			
		Primary	*p* Value	Secondary	*p* Value
**All journals**	2127	1667		1440	
Academic	255 (10.6%)	158 (9.5%)	0.001	148 (10.3%)	0.259
Industry	178 (8.4%)	127 (7.6%)		121 (8.4%)	
Organizations	1.189 (55.9%)	948 (56.9%)		800 (55.6%)	
None disclosed	299 (14.1%)	236 (14.2%)		197 (13.7%)	
None	236 (11.1%)	198 (11.9%)		174 (12.1%)	
***AJCN***	1020	786		676	
Academic	96 (9.4%)	67 (8.5%)	0.320	65 (9.6%)	0.941
Industry	82 (8.0%)	61 (7.8%)		54 (8.0%)	
Organizations	741 (72.6%)	578 (73.5%)		491 (72.6%)	
None disclosed	19 (1.9%)	14 (1.8%)		11 (1.6%)	
None	82 (8.05)	66 (8.4%)		55 (8.1%)	
***EJCN***	720	561		474	
Academic	81 (11.3%)	58 (10.3%)	0.036	50 (10.5%)	0.467
Industry	59 (8.2%)	39 (7.0%)		40 (8.4%)	
Organizations	331 (46.0%)	267 (47.65)		222 (46.8%)	
None disclosed	225 (31.3%)	175 (31.25)		143 (30.2%)	
None	24 (3.3%)	22 (3.95)		19 (4.0%)	
***Clin Nutr ESPEN***	387	320		290	
Academic	48 (12.4%)	33 (10.3%)	0.022	33 (11.4%)	0.992
Industry	37 (9.6%)	27 (8.4%)		27 (9.3%)	
Organizations	117 (30.2%)	103 (32.2%)		87 (30.0%)	
None disclosed	55 (14.2%)	47 (14.7%)		43 (14.8%)	
None	130 (33.6%)	110 (34.4%)		100 (34.5%)	

AJCN, American Journal of Clinical Nutrition; Clin Nutr ESPEN, Clinical Nutrition ESPEN; EJCN, European Journal of Clinical Nutrition.

**Table 3:** Positive findings in the primary and secondary outcomes of studies according to the secondary funding source.

Funding Source	N	Outcomes			
		Primary	*p* Value	Secondary	*p* Value
**All journals**	2127	1667		1440	
Academic	151 (7.1%)	118 (7.1%)	0.673	97 (6.7%)	0.002
Industry	116 (5.5%)	85 (5.1%)		82 (5.7%)	
Organizations	831 (39.1%)	653 (39.2%)		541 (37.6%)	
None disclosed	346 (16.3%)	272 (16.3%)		227 (15.8%)	
None	683 (32.15)	539 (32.3%)		493 (34.2%)	
***AJCN***	1020	786		676	
Academic	87 (8.5%)	67 (8.5%)	0.815	52 (7.7%)	0.291
Industry	73 (7.2%)	54 (6.9%)		49 (7.2%)	
Organizations	575 (56.4%)	450 (57.3%)		377 (55.8%)	
None disclosed	63 (6.2%)	48 (6.1%)		40 (5.9%)	
None	222 (21.8%)	167 (21.2%)		158 (23.4%)	
***EJCN***	720	561		474	
Academic	40 (5.6%)	29 (5.2%)	0.614	27 (5.7%)	0.384
Industry	31 (4.3%)	21 (3.7%)		21 (4.4%)	
Organizations	196 (27.2%)	155 (27.6%)		126 (26.6%)	
None disclosed	228 (31.7%)	177 (31.6%)		144 (30.4%)	
None	225 (31.3%)	179 (31.9%)		156 (32.9%)	
***Clin Nutr ESPEN***	387	320		290	
Academic	24 (6.2%)	22 (6.9%)	0.674	18 (6.2%)	0.102
Industry	12 (3.1%)	10 (3.1%)		12 (4.1%)	
Organizations	60 (15.5%)	48 (15.0%)		38 (13.1%)	
None disclosed	55 (14.2%)	47 (14.7%)		43 (14.8%)	
None	236 (61.0)	193 (60.3%)		179 (61.7%)	

AJCN, American Journal of Clinical Nutrition; Clin Nutr ESPEN, Clinical Nutrition ESPEN; EJCN, European Journal of Clinical Nutrition.

**Table 4:** Positive findings in the primary and secondary outcomes of studies according to the age of participants.

Age of Participants	N	Outcomes			
		Primary	*p* Value	Secondary	*p* Value
**All journals**	2127	2114		1440	
Adults	1651 (77.6%)	1293 (77.6%)	0.924	1140 (79.2%)	0.203
Minors	333 (15.7%)	260 (15.6%)		210 (14.6%)	
Unspecified sample age	35 (1.6%)	27 (1.6%)		19 (1.3%)	
Mixed adults and minors	108 (5.1%)	87 (5.2%)		71 (4.9%)	
***AJCN***	1020	786		676	
Adults	807 (79.1%)	619 (78.8%)	0.753	549 (81.2%)	0.020
Minors	154 (15.1%)	120 (15.3%)		90 (13.3%)	
Unspecified sample age	8 (0.8%)	6 (0.8%)		2 (0.3%)	
Mixed adults and minors	51 (5.0%)	41 (5.2%)		35 (5.2%)	
***EJCN***	720	561		474	
Adults	540 (75.0%)	428 (76.3%)	0.273	358 (75.5%)	0.677
Minors	127 (17.6%)	93 (16.6%)		82 (17.3%)	
Unspecified sample age	14 (1.9%)	9 (1.6%)		7 (1.5%)	
Mixed adults and minors	39 (5.4%)	31 (5.5%)		27 (5.7%)	
***Clin Nutr ESPEN***	387	320		290	
Adults	304 (78.6%)	246 (76.9%)	0.366	233 (80.3%)	0.048
Minors	52 (13.4%)	47 (14.7%)		38 (13.1%)	
Unspecified sample age	13 (3.4%)	12 (3.8%)		10 (3.4%)	
Mixed adults and minors	18 (4.7%)	15 (4.7)		9 (3.1%)	

AJCN, American Journal of Clinical Nutrition; Clin Nutr ESPEN, Clinical Nutrition ESPEN; EJCN, European Journal of Clinical Nutrition.

**Table 5:** Positive findings in the primary and secondary outcomes of studies according to the existence of a pre-published protocol.

Published Protocols	N	Outcomes			
		Primary	*p* Value	Secondary	*p* Value
**All journals**	797	580		514	
Yes	566 (71.0%)	417 (71.9%)	0.427	377 (73.3%)	0.203
No	231 (29.0%)	163 (28.1%)		137 (26.7%)	
**SRs/** **ΜAs/** **ΜR**	210	152		125	
Yes	68 (32.4%)	51 (33.6%)	0.544	44 (35.2%)	0.428
No	142 (67.6%)	101 (66.4%)		81 (64.8%)	
**RCTs**	587	428		389	
Yes	498 (84.8%)	366 (85.5%)	0.436	333 (85.6%)	0.613
No	89 (15.2%)	62 (14.5%)		56 (14.4%)	

MA, meta-analysis; MR, meta-research; RCT, randomized controlled trial; SR, systematic review.

**Table 6:** Positive findings in the primary and secondary outcomes of studies adopting the energy adjustment criteria.

Adjustment for the EI of Participants	N	Outcomes			
		Primary	*p* Value	Secondary	*p* Value
**Items published in all journals**	531	421		374	
Yes	181 (34.1%)	137 (32.5%)	0.095	130 (34.8%)	0.719
No	350 (65.9%)	284 (67.5%)		244 (65.2%)	
**Cohort studies**	302	234		204	
Yes	114 (37.7%)	79 (33.8%)	0.006	75 (36.8%)	0.638
No	188 (62.3%)	155 (66.2%)		129 (63.2%)	
**Cross-sectional studies**	229	187		170	
Yes	67 (29.3%)	58 (31.0%)	0.284	55 (32.4%)	0.138
No	162 (70.7%)	129 (69.0%)		115 (67.6%)	

EI, energy intake.

## Data Availability

All data are presented within the manuscript text and tables.
